# Bacteriophage MS2 As a Tool for Targeted Delivery in Solid Tumor Chemotherapy

**DOI:** 10.32607/20758251-2019-11-2-98-101

**Published:** 2019

**Authors:** E. F. Kolesanova, M. V. Melnikova, T. N. Bolshakova, E. Yu. Rybalkina, I. G. Sivov

**Affiliations:** Institute of Biomedical Chemistry, Pogodinskaya Str. 10, bld. 8, Moscow, 119121, Russia; N.F. Gamaleya Federal Research Center of Epidemiology and Microbiology, Gamalei Str. 18, Moscow, 123098, Russia; Institute of Carcinogenesis, Federal National Medical Research Center of Oncology, Kashirskoe sh. 23 , Moscow, 115478, Russia; Biotechnologiya, Ltd., Efremova Str. 20, Moscow, 119048, Russia

**Keywords:** bacteriophage MS2, iRGD peptide, thallium (I) ions, targeted therapy, breast cancer

## Abstract

Bacteriophage MS2 was employed for targeted delivery of an apoptosis-inducing agent, Tl+, into a tumor tissue. The targeted delivery was ensured by iRGD peptide, a ligand of integrins presumably located on the surface of endotheliocytes of the tumor tissue neovasculature and certain tumor cells. The synthesized peptide was conjugated to MS2 capsid proteins. Tl+ ions from TlNO3 penetrated the phage particles and tightly bound to phage RNA. Peptide-modified MS2 preparations filled with Tl+ caused cell death in two types of cultivated human breast cancer cells and effected necrosis of these tumor xenografts in mice. Neither peptide-conjugated bacteriophage MS2 without Tl+ nor the phage filled with Tl+ but without the peptide or the same phage with the non-conjugated peptide in solution produced such effects. The preparation exhibited no acute toxicity at a therapeutic dose.

## INTRODUCTION

Recently, efforts by researchers involved in the development of anti-tumor drugs have focused on targeted therapeutic agents based both on novel and already-known cytostatic drugs [1]. The use of nanocontainers (liposomes, micelles, polymer nanoparticles, virus-like particles, and viruses) modified with specific ligands filled with a drug is considered the most efficient delivery method [2]. However, these innovative delivery methods do not solve the problem of cancer multidrug resistance, which has the potential to undermine all previous efforts to enhance drug efficacy [3].

It has been demonstrated that Tl^+^ ions exhibit strong cytotoxic activity and inhibit the cancer drug resistance-associated protein that acts as an efflux pump [4]. Incorporation of Tl^+^ into a “non-leaking” nanosized container equipped with a targeted delivery system could allow one to develop an efficient tool for tumor destruction, while the overall toxicity of Tl^+^ can be significantly mitigated. In the 1980s, Tl^+^ ions were successfully entrapped in cowpox virus particles [5]. The entrapment mechanism involved the formation of a strong conjugate between Tl^+^ and viral RNA [6]. The bacteriophage MS2 selected as a nanocontainer can reproduce itself only in *Escherichia coli *cells that carry F-pili and are neither human symbionts nor pathogens [7]. The delivery direction was ensured via conjugation of phage capsid proteins and the (Gly)3-iRGD peptide carrying the cycloSS-(CRGDKGPDC) (iRGD) moiety, which is responsible for binding to integrins that predominantly localize on the outer membranes of endothelial cells of the pathological neovasculature of solid tumors and on a number of tumor cells [8]. In this study, we experimentally tested the effectiveness of Tl^+^-filled bacteriophage MS2 carrying a targeting peptide as a candidate antitumor agent.

## EXPERIMENTAL

The procedure used to prepare bacteriophage MS2 was described earlier in [9]. The number of plaque-forming units (PFUs) per milliliter of the phage preparation was identified by agar overlay assay.

(Gly)3-iRGD peptide was prepared by automated solid-phase synthesis using 9-fluorenylmethoxycarbonyl amino acids (ChemPep, USA) on a 433A peptide synthesizer (Applied Biosystems) through the FastMoc method. The S–S bridge was formed by oxidation with I2 [10]. The peptide was purified by reversed-phase HPLC (YMC-Triart C18 column, 21 × 250 mm, 10.0 μm, Switzerland; Agilent 1100 working station, Agilent, USA), elution by CH_3_CN (BioSolve, Israel) concentration gradient in water containing 0.1% acetic acid. According to the data obtained by analytical reversed-phase HPLC (YMC-Triart C18 column, 2.1 × 50 mm, 2.0 μm, Agilent 1200 working station) with UV and mass-spectrometry detection, purity of the peptide preparation was ≥ 95%.

(Gly)_3_-iRGD peptide was conjugated to bacteriophage MS2 capsid proteins using a homobifunctional reagent dimethyl adipimidate (DMAI, Sigma, USA) at a phage protein : peptide : DMAI molar ratio of 1 : 20 : 80, using the procedure described in [11]. The bacteriophage was separated from the excess reagents via precipitation with a 25% polyethylene glycol 6000 solution (Dia-M, Russia) containing 1 M NaCl. The precipitated bacteriophage was suspended in deionized water.

The bacteriophage was filled with Tl**+ **using TlNO_3_ (Sigma-Aldrich, USA). The peptide-conjugated bacteriophage MS2 (iRGD-MS2) (1011 PFUs) was incubated in 3 ml of a 0.5 μM TlNO_3_ solution (5 h at 38°C), followed by precipitation according to the procedure described above and dialysis against phosphate buffered saline (0.14 M NaCl, 0.01 M sodium phosphate, pH 7.4).

The amounts of Tl^+^ ions both inside and outside the virions (in the medium) were determined using the procedure described in [12]. A suspension of bacteriophage particles filled with Tl^+^ was centrifuged for 10 min at 5,000 rpm to remove the thallium salt precipitate, diluted with 50 mM Tris-HCl buffer (pH 9.0) until a nominal concentration of 108 PFUs/ml, and denatured by heating with RNase in 0.05% SDS at +70°C for 30 min. Quenching of 1,3,6,8-pyrene tetrasulfonic acid fluorescence by Tl^+^ ions was then recorded (excitation wavelength, 340 nm; emission wavelength, 465 nm) on an UV-1900 spectrofluorometer (BOC Sciences APP, USA). A calibration curve showing the dependence between the fluorescence quenching degree and [Tl^+^] was used to calculate the content of Tl in the bacteriophage preparation. The Tl content in the buffer solution after dialysis was determined without pre-denaturation.

The cytotoxic effect of iRGD-MS2-Tl^+^ on the cell cultures was studied using MCF-7 (hormone-dependent breast cancer) and MDA-MB-231 (hormone-independent breast cancer) cell lines. The cells were cultured in a serum-free medium (MSC1 Pan BioTech) and in the same medium supplemented with 5% fetal calf serum. The iRGD-MS2-Tl^+^ preparation was added in 10-fold dilutions, starting with a concentration of 108 PFU/ml. The iRGD-MS2 preparation (the peptide-conjugated bacteriophage without Tl^+^) was used as a control. Dead cells were counted after staining with Evans blue. The antitumor effect of the iRGD-MS2-Tl^+^ preparation was tested in nude mice with MCF-7 or MDA-MB-231 cancer cell-derived xenografts. The mice were injected with 105–106 MCF-7 or MDA-MB-231 cells intradermally. Fourteen days later, the mice in the experimental groups received 200 μl of a suspension containing iRGD-MS2-Tl^+^ at a dose corresponding to 108 PFU/kg intraperitoneally during 10 days (once per day). Mice in the control group were injected with iRGD-MS2, MS2-Tl^+^, or MS2-Tl^+^ + iRGD (2 μg/kg in solution) of the MS2 dosage equal to the iRGD-MS2-Tl^+^ doses for experimental animals, and in the same volume of the solution. Each experimental and control group consisted of 11 animals. The necrotic activity of the preparation was determined as a ratio between the area of necrosis tissue and the total area of the tumor by analyzing digital images of histologic sections recorded using a ScanScope CS2 scanner 12 days after the last injection of bacteriophage preparations.

Acute toxicity of the iRGD-MS2-Tl^+^ preparation was preliminarily studied on 10 female Wistar Kyoto (WKY) rats (weight, 200–250 g). The rats were housed under the conditions of 12-hour light and 12-hour dark cycle and given *ad libitum *access to a standard laboratory diet and water. The animals received a single intradermal injection of the preparation (108 PFU/animal, 500 μl). The state of the animals was monitored during three weeks post-injection.

Experiments on animals were carried out in compliance with the International Guidelines of the European Convention for the Protection of Vertebrate Animals used for Experimental and Other Scientific Purposes, and the principles of Good Laboratory Practice (GLP) approved by Degree no. 267 of the Ministry of Health of the Russian Federation dated June 19, 2003.

## RESULTS AND DISCUSSION

MS2 preparations containing Tl^+^ in the amount of 2.0 × 10-9 g-eq thallium per 108 PFUs were obtained by incubating the bacteriophage MS2 (both modified and unmodified with iRGD peptide) in the medium with TlNO_3_. The Tl^+^ content per PFU was 2.0 × 10-17 g-eq (~ 4 femtogram per PFU; i.e., 400 ng per 108 PFU). No Tl^+^ ions were detected in the buffer solution used for dialysis of Th+-filled bacteriophage, which indicates that Th+ is tightly bound to phage RNA inside MS2 particles.

**Figure 1:**
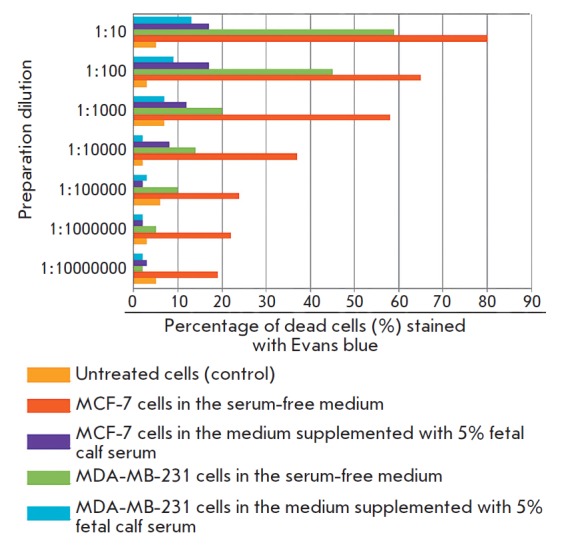
The toxic effect of the iRGD-MS2-Tl^+^ preparation on tumor cell cultures (cell death, %)

*Figure 1* demonstrates that the iRGD-MS2-Tl**+** preparation had a cytotoxic effect on hormone-dependent and hormone-independent breast cancer cells in the serum-free medium. For hormone-dependent BC (MCF-7 cells), ED50 of the iRGD-MS2-Tl^+^ preparation was slightly lower than 105 PFU/ml of the culture broth, while the cytotoxic effect of the preparation was statistically significant compared to the control specimen, up to a concentration of 10 PFU/ml. Hormone-independent breast cancer (MDA-MB-231) cells were more resistant to the preparation: for these cells, ED50 in a serum-free medium was 106–107 PFU/ml, while the cytotoxic effect of the preparation was statistically significant compared to the control specimen, up to a concentration of 104 PFU/ml. The cytotoxic activity of iRGD-MS2-Tl^+^ was much weaker in the serum-containing medium, which may be an indication that serum components and iRGD-MS2-Tl^+^ particles compete for penetration into the cells.

**Fig. 2:**
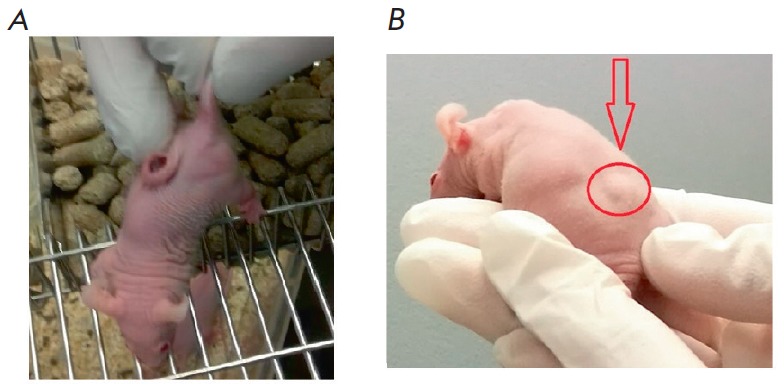
MDA-MB-231 tumor in xenograft mice before (A) and after (B) treatment with a iRGD-MS2-Tl**+ **preparation

**Fig. 3:**
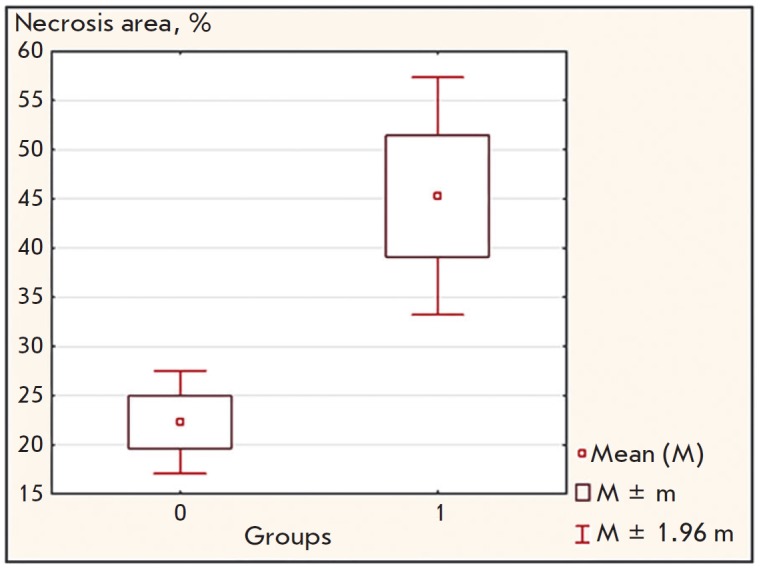
Area of tumor tissue necrosis in mice xenograft of human BC. Group 1 – experimental animals that received a iRGD-MS2-Tl**+ **preparation; group 0 – control animals that received iRGD-MS2, or MS2-Tl**+**, or MS2-Tl^+^ with a non-conjugated iRGD peptide (in a solution)

In mice with MCF-7 and MDA-MB-231 xenografts, the tumor volume was reduced 12 days following the injections of the iRGD-MS2-Tl^+^ preparation compared to that in the control animals (*Fig. 2*). The necrotizing effect of iRGD-MS2-Tl^+^ on the corresponding tumors was demonstrated histochemically. *Figure 3* shows that the iRGD-MS2-Tl^+^ preparation was more efficient in causing tumor tissue necrosis (*p* < 0.05) than peptide-conjugated phage preparations without Tl^+^ ions, Tl^+^-filled phage preparations without the peptide, or Tl^+^-filled phage preparations containing the non-conjugated peptide in the solution.

Evaluation of acute toxicity of the iRGD-MS2-Tl^+^ preparation in Wistar Kyoto (WKY) rats demonstrated that a single-dose injection of iRGD-MS2-Tl^+^ (108 PFU/animal; i.e., 1.6–2.0 μg Tl/kg) caused death in none of the animals after three weeks of follow-up. No noticeable changes in animal behavior were revealed. The total therapeutic dose of Tl^+^ (4 μg/kg) was 5,000- fold lower than its LD_50_ (20 mg/kg).

## CONCLUSIONS

Targeted delivery of ions of a toxic metal to a tumor neovasculature using phage display based on iRGD-MS2-Tl**^+^** particles causes efficient degradation of the entire tumor mass, while the risk of overall toxicity is significantly reduced. Therefore, it is reasonable to recommend conducting preclinical trials of iRGD-MS2-Tl**^+^** in order to develop a preparation which can potentially be further used to treat breast cancer. Since the iRGD peptide ligand interacts with a_ν_b_3_ and a_ν_b_5_ integrins on the surface of endothelial cells in the pathological vasculature [8], this drug may be efficient against other solid tumors characterized by intensive pathological neoangiogenesis.

## Abbreviations

BC: breast cancer
DMAI: dimethyl adipimidate
ED50: effective dose (the dose that causes an effect equal to 50% of the maximal one)
HPLC: high-performance liquid chromatography
LD50: dose of a substance lethal to 50% tested animals
PFU: plaque-forming units
SDS: sodium dodecyl sulfate
